# Characterization of renal artery variation in patients with clear cell renal cell carcinoma and the predictive value of accessory renal artery in pathological grading of renal cell carcinoma: a retrospective and observational study

**DOI:** 10.1186/s12885-023-10756-y

**Published:** 2023-03-25

**Authors:** Dingyang Lv, Huiyu Zhou, Fan Cui, Jie Wen, Weibing Shuang

**Affiliations:** 1 Department of Urology, First Hospital of Shanxi Medical University, No. 85 Jiefang South Road, Yingze District, Taiyuan, Shanxi Province China; 2 First Clinical Medical College of Shanxi Medical University, No. 56 Xinjian South Road, Yingze District, Taiyuan, Shanxi Province China

**Keywords:** Renal cell carcinoma, Renal artery variation, Accessory renal artery, Fuhrman grade

## Abstract

**Objective:**

To explore the characteristics of renal artery variation in patients with renal cell carcinoma and to evaluate the predicting value of accessory renal artery in the pathological grading of renal cell carcinoma.

**Methods:**

The clinicopathological data of patients with clear cell renal cell carcinoma diagnosed in the Department of Urology of the First Hospital of Shanxi Medical University from September 2019 to March 2023 were retrospectively analyzed. All patients underwent visual three-dimensional model reconstruction from computed tomography images. All kidneys were divided into two groups: the affected kidney and the healthy kidney, and the incidence of renal artery variation in the two groups was analyzed. Then, according to the existence of accessory renal artery in the affected kidney, the patients were divided into two groups, and the relationship between accessory renal artery and clinicopathological features of patients with clear cell renal cell carcinoma was analyzed. Finally, univariate and multivariate logistic regression analyses were performed to determine the predictors of Fuhrman grading of clear cell renal cell carcinoma, and the predictive ability of the model was evaluated by the receiver operating characteristic curve.

**Results:**

The incidence of renal artery variation and accessory renal artery in the affected kidney was significantly higher than them in the healthy kidney. The patients with accessory renal artery in the affected kidney had larger tumor maximum diameter, higher Fuhrman grade and more exophytic growth. The presence of accessory renal artery on the affected kidney and the maximum diameter of tumor are independent predictors of high-grade renal cell carcinoma. The receiver operating characteristic curve suggests that the model has a good predictive ability.

**Conclusion:**

The existence of accessory renal artery on the affected kidney may be related to the occurrence and development of clear cell renal cell carcinoma, and can better predict Fuhrman grade of clear cell renal cell carcinoma. The finding provides a reference for the future diagnostic evaluation of RCC, and provides a new direction for the study of the pathogenesis of RCC.

## Introduction

Renal cell carcinoma (RCC) is one of the common malignant tumors of the urinary system, and it ranks third for incidence, after prostate and bladder cancer, accounting for 2%-3% of adult malignant tumors [1]. Among the histological subtypes of RCC, clear cell RCC (ccRCC) is the most common one, accounting for 70–80% of cases [2]. About 16% of the patients present with metastatic renal cell carcinoma at their first visits. In recent years, the use of advanced imaging technologies has promoted early diagnosis of RCC. The incidence of RCC is on the rise, however, its etiology is still poorly understood. At present, the main surgical approaches of RCC are radical nephrectomy and partial nephrectomy, and there are still greater risks of recurrence and metastasis after surgery [3–5]. Therefore, it is necessary to further explore the mechanisms underlying the occurrence and development of RCC.

The evaluation of tumors has been one of the core tasks in clinical diagnosis and treatment of diseases, especially for ccRCC, a malignant tumor with poor prognosis. Tumor grade describes the degree of differentiation of tumor cells relative to normal cells, and it is a reliable indicator of tumor growth and metastasis. Studies have pointed out that the pathological grade of RCC is one of the major predictors of prognosis. Usually, the higher the grade, the worse the prognosis [6]. Fuhrman grading system has been widely used in pathological grading of RCC, and it is established based on the evaluation of several nuclear characteristics: nuclear size, nuclear shape and nucleolar prominence. According to these characteristics, tumors are classified into four different grades (I-IV). Grades I and II are considered to be low-grade tumors with good prognosis, while grades III and IV are considered to be high-grade tumors with poor prognosis [7]. Yu et al. [8] found that the prognosis of patients with low-grade RCC is relatively better than that of high-grade patients, the safety of partial nephrectomy is higher, and the incidence of postoperative complications is lower. Tabourin et al. [9] reported that Fuhrman grade > 2 is one of the important risk factors for postoperative recurrence of RCC. However, Fuhrman grade can only be determined by postoperative pathology examination, and it is still challenging to obtain them timely and effectively before surgical treatment of RCC.

Studies have found that the incidence of renal artery variation (RAV) in patients with RCC ranges between 17.5% and 25.1%, which is higher than 14.01% in the general population [10, 11]. And in the clinical work, we often found that the affect kidney of patients with renal cell carcinoma was often accompanied by accessory renal artery. Therefore, we speculate that there is a certain relationship between the occurrence and development of RCC and RAV. To confirm this hypothesis, we observed the characteristics of RAV in patients with RCC based visual three-dimensional (3D) models reconstructed from computed tomography (CT) images, and explored the correlation between accessory renal artery (ARA) and clinicopathological features of patients, as well as the predictive factors of tumor pathological grade. The findings are expected to enhance our understanding of the mechanism of occurrence and development and treatment of RCC.

## Materials and methods

### Data collection

We retrospectively collected the clinical data of patients with ccRCC in the Department of Urology of First Hospital of Shanxi Medical University from September 2019 to March 2023. The clinicopathological features of patients included sex, age, body mass index (BMI), tumor location, maximum tumor diameter, tumor growth pattern, Fuhrman grade, and hypertension. Inclusion criteria were as follows: (1) patients had unilateral single tumor; (2) patients underwent plain and enhanced urinary CT scans or abdominal and pelvic CT scans, as well as visual 3D model reconstruction from CT images; and (3) ccRCC was confirmed by renal puncture or postoperative pathology report. Patients with incomplete data were excluded. This study complied with the Declaration of Helsinki. The study was approved by the ethics committee of the First Hospital of Shanxi Medical University (Ethical code: [2021] K048). The written informed consent was waived by the First Hospital of Shanxi Medical University of the Institutional Review Board.

**Fig. 1:**
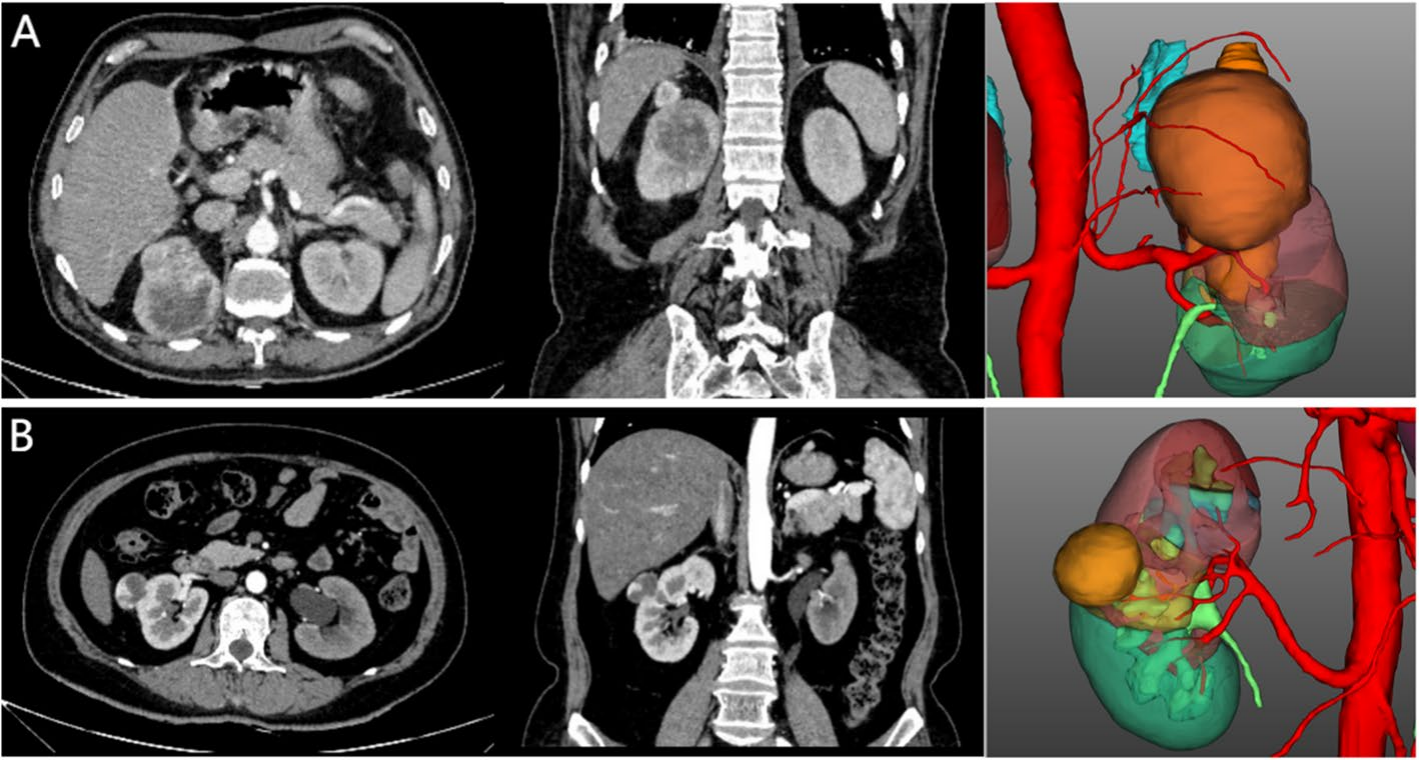
Representative CT images and reconstructed 3D models for one male patient and one female patient. Legends: **A** Male, 70 years old, right renal tumor, the maximum tumor diameter was 7.5 cm, there were 2 ARA and 1 branch of renal artery before the renal hilum. **B** Female, 50 years old, right renal tumor, the maximum tumor diameter was 3.0 cm, there is 1 ARA

All patients underwent CT scanning, and CT images were used for 3D model reconstruction. The typical reconstructed 3D models are shown in Fig. 1.

### Classification of RAV

There are different classification methods of RAV. ARA and branches of renal artery before renal hilum are the most clinically significant, which have attracted much attention from scholars. According to the study of Satyapal et al. [12], we defined ARAs as one or more arteries originating from thoracic aorta, abdominal aorta or its branches (superior and inferior mesenteric artery, common iliac artery, etc.), except renal artery. Branches of renal artery before renal hilum are defined as one or more branches from the trunk of the renal artery before entering the renal hilum. The number of renal arteries, ARA and branches of renal artery before renal hilum were recorded by observing CT images and reconstructed visual 3D model, and the incidence of all sorts of variations was calculated.

### Pathological grading of ccRCC

The pathological grading was conducted by the pathologists of our hospital using the Fuhrman grading system. Patients with ccRCC were grouped into a low-grade group (Fuhrman grades I and II) and a high-grade group (Fuhrman grades III and IV).

### Statistical analysis

Continuous variables were expressed as means ± standard deviation and analyzed by the Student’s t-test, while categorical variables were presented as counts (percentages) and analyzed by the Chi-squared test. All kidneys were divided into two groups: the affected kidney and the healthy kidney. The incidence of RAV in the two groups was analyzed. Then, according to the presence or absence of ARA on the affected kidney, the patients were divided into two groups, and the relationship between ARA and clinicopathological features of patients with ccRCC was analyzed. Finally, the presence or absence of ARA on the affected kidney, sex, age, body mass index (BMI), tumor location, maximum tumor diameter, tumor growth pattern, and hypertension were included in univariate and multivariate logistic regression analysis to determine the independent predictors of tumor pathological grade. The receiver operating characteristic (ROC) curve and AUC were used to evaluate its predictive ability. All statistical analyses were performed using SPSS version 26 (IBM Corporation). Differences were considered statistically significant at *P* < 0.05.

## Results

### Clinical characteristics of patients

A total of 99 patients with ccRCC were enrolled in the study. The baseline characteristics of the patients are summarised in Table 1. The mean age of the patients was 57.99 ± 9.99 years, and there were 54 males (54.5%) and 46 patients with hypertension (46.5%). The mean BMI was 25.46 ± 3.21 kg/m2, and the mean maximum tumor diameter was 4.34 ± 2.73 cm. The inferior pole of kidney (43.4%) was the major site where ccRCC was located. The high-grade ccRCC accounted for 26.3%, and 80.8% of ccRCC showed an exophytic growth pattern.

**Table 1:** The baseline characteristics of the patients

Variables	Value
N	99
Age, years	57.99 ± 9.99
Sex, n (%)	
Male	54 (54.5)
Female	45 (45.5)
BMI, kg/m^2^	25.46 ± 3.21
Maximum tumor diameter, cm	4.34 ± 2.73
Tumor location, n (%)	
Superior pole of kidney	26 (26.2)
Middle pole of kidney	30 (30.3)
Inferior pole of kidney	43 (43.4)
Tumor growth pattern, n (%)	
Exogenous	80 (80.8)
Endogeny	19 (19.2)
Hypertension, n (%)	
Yes	46 (46.5)
No	53 (53.5)
Fuhrman grade, n (%)	
High	26 (26.3)
Low	73 (73.7)

Continuous variables are expressed as mean ± standard deviation and categorical variables as counts (percentages)

### Correlation between RAV and ccRCC

All the kidneys were divided into two groups: the affected kidney and the healthy kidney. The incidence of RAV in the affected kidney was significantly higher than that in the healthy kidney (*P* = 0.003). Furthermore, we divided RAV into ARA and the branches of renal artery before renal hilum. The incidence of ARA in the affected kidney was significantly higher than that in the healthy kidney (*P* = 0.000, Table 2), but there was no significant difference in the incidence of the branches of renal artery before renal hilum between the two groups (*P* = 0.144, Table 2).

**Table 2:** Correlation between RAV and ccRCC

Group	RAV		ARA		Branches of renal artery before the renal hilum	
	Yes	No	Yes	No	Yes	No
The affected kidney, n (%)	57 (57.6)	42 (42.4)	42 (42.4)	57 (57.6)	30 (30.3)	69 (69.7)
The healthy kidney, n (%)	36 (36.4)	63 (63.6)	19 (19.2)	80 (80.8)	21 (21.2)	78 (78.8)
*χ*^*2*^ value	8.942		12.533		2.139	
*P* value	0.003		0.000		0.144	

### Correlation between ARA and clinicopathological features of patients with ccRCC

The patients were divided into two groups according to the presence or absence of ARA in the affected kidney. There was no significant difference in sex, age, BMI, tumor location and hypertension between the two groups. However, compared to the group without ARA in the affected kidney, the group with ARA in the affected kidney had a larger tumor diameter (*P* = 0.000, Table 3) and a higher Fuhrman grade (*P* = 0.000, Table 3) and exhibited more exophytic growth (*P* = 0.000, Table 3).

**Table 3:** Correlation between ARA and clinicopathological features of patients with ccRCC

Variables	Group with ARA in the affected kidney (*n* = 41)	Group without ARA in the affected kidney (*n* = 58)	*P* value
Sex, n (%)			0.503
Male	24 (58.54)	30 (51.72)	
Female	17 (41.46)	28 (48.28)	
Age, years	59.14 ± 10.46	57.57 ± 9.38	0.711
BMI, kg/m^2^	24.85 ± 2.52	25.52 ± 3.63	0.855
Hypertension, n (%)			0.228
Yes	22 (53.66)	24 (41.38)	
No	19 (46.34)	34 (58.62)	
Maximum tumor diameter, cm	5.61 ± 2.86	3.11 ± 1.46	0.000
Tumor location, n (%)			0.449
Superior pole of kidney	13 (31.70)	13 (22.41)	
Middle pole of kidney	10 (24.39)	20 (34.48)	
Inferior pole of kidney	18 (43.90)	25 (43.10)	
Tumor growth pattern, n (%)			0.000
Exophytic	39 (95.12)	41 (70.69)	
Endogenetic	2 (4.88)	17 (29.31)	
Fuhrman grade, n (%)			0.000
High	20 (48.78)	6 (10.34)	
Low	21 (51.22)	52 (89.66)	

### ARA predicts Fuhrman high-grade ccRCC in patients

**Fig. 2:**
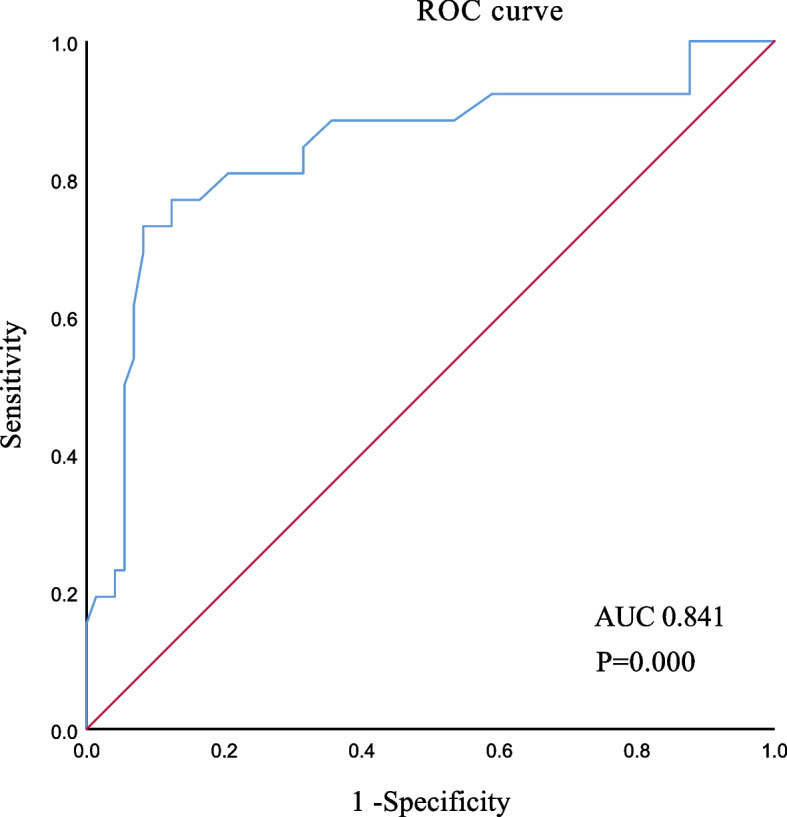
ROC curve. Legends: The AUC of the multivariate model was 0.841 (95%CI, 0.739–0.942; *P* = 0.000), indicating that this model had high predictive ability

To explore the association between ARA and Fuhrman grade of ccRCC, Logistic regression analysis was performed on each variable for the univariate model (Table 4). In the univariate model, the presence of ARA on the affected kidney (OR, 8.254; 95%CI, 2.907–23.437; *P* = 0.000) and maximum tumor diameter (OR, 1.700; 95%CI, 1.300–2.224; *P* = 0.000) were found to be significantly associated with high-grade ccRCC. Logistic regression was then performed for these two variables for the multivariate model (Table 4). In the multivariate model, the presence of ARA on the affected kidney (OR, 4.242; 95%CI, 1.135–13.331; *P* = 0.013) and maximum tumor diameter (OR, 1.536; 95%CI, 1.170–2.017; *P* = 0.002) remained significant predictors of high-grade ccRCC. As shown in Fig. 2, the AUC of the multivariate model was 0.841 (95%CI, 0.739–0.942; *P* = 0.000), indicating that this model had high predictive ability.

**Table 4:** Univariate and multivariate models for predicting high-grade ccRCC

Variables	Univariate analysis			Multivariate analysis		
	OR	95%CI	*P* value	OR	95%CI	*P* value
Sex, male/female	1.838	0.726–4.654	0.199			
Age, years	1.017	0.971–1.066	0.477			
BMI, kg/m^2^	0.954	0.823–1.106	0.533			
Hypertension, yes/no	0.796	0.323–1.965	0.621			
ARA on the affected kidney, presence/absence	8.254	2.907–23.437	0.000	4.242	1.350–13.331	0.013
Tumor location			0.113			
Superior pole of kidney	reference	reference	reference			
Middle pole of kidney	0.246	0.066–0.918	0.037			
Inferior pole of kidney	0.619	0.220–1.741	0.364			
Maximum tumor diameter, cm	1.700	1.300–2.224	0.000	1.536	1.170–2.017	0.002
Tumor growth pattern, exophytic/endogenetic	3.643	0.780–17.013	0.100

## Discussion

In general, each kidney of a normal adult is supplied by a single artery. However, in the embryonic stage, the developing mesonephros, metanephros, suprarenal glands, and gonads are supplied by nine pairs of lateral mesonephric arteries arising from the dorsal aorta. These nine pairs of arteries are divided into three groups: cranial (1st and 2nd pair), middle (3rd–5th pair), and caudal (6th–9th) [13]. The renal artery develops from one of the pairs in the middle group. With the gradual upward movement of the metanephros, renal artery variantions can be formed if the remaining arteries are not completely degenerated. The incidence of RAV varies widely worldwide. The incidence of RAV in the Chinese population is 14.5% [14]. In the Greek population, the incidence of RAV is 11.2% [15]. In Colombia, nearly a third of its population has one additional renal artery, and about 3% of the same population has two additional renal arteries, most of which reach the kidney through its hilar region [16]. In addition, the incidence of RAV in patients with RCC is higher than that in normal people [10, 11], suggesting that the occurrence and development of RCC may be related to RAV. To elucidate the relationship between RAV and RCC, we divided RAV into two categories: ARA and the branches of renal artery before renal hilum. It is found that the incidence of ARA in the affected kidney was significantly higher than that in the healthy kidney, indicating that ARA may play an important role in the occurrence and development of RCC. At present, there is no evidence showing the relationship between ARA and RCC.

In order to explore the correlation between RAV and ccRCC, we divided all kidneys into two groups: the affected kidney and the healthy kidney, and found that the incidence of RAV in the affected kidney was significantly higher than that in the healthy kidney. Furthermore, we found that the incidence of ARA in the affected kidney was significantly higher than that in the healthy side of kidney, but we did not find significant difference in the incidence of branches of renal artery before renal hilum between the two groups. This result indicated that the significant difference in the incidence of RAV between the two groups is caused by the difference of in the incidence of ARA.

Furthermore, we found that the group with ARA in the affected kidney had a larger tumor maximum diameter and higher Fuhrman grades, and ccRCC showed more exophytic growth. The reason may be that the blood supply of the tumor with ARA in the affected kidney is so abundant that the tumor grows faster, which leads to high tumor invasiveness and a high degree of malignancy. Previous studies have shown that tumor growth patterns can predict ccRCC pathological grade [17–20] and high-grade ccRCC mostly show exophytic growth. Our study found that most of the tumors with ARA in the affected kidney showed exophytic growth and higher Fuhrman grades, which indirectly confirmed the above conclusion.

Previous studies have shown that tumor diameter, BMI and tumor growth pattern can well predict the pathological grade of RCC. Zhu et al. [21] analyzed 186 patients with RCC and found that tumor size (OR, 1.91; 95%CI, 1.01–3.60; *P* = 0.047) was one of the independent risk factors for high-grade RCC. Hu et al. [22] found that maximum tumor diameter was a significant predictor of high-grade ccRCC in both univariate (OR, 1.136; 95%CI, 1.003–1.287; *P* = 0.045) and multivariate (OR, 1.159; 95%CI, 1.018–1.320; *P* = 0.025) models. Our study obtained the same results through univariate and multivariate logistic regression analysis. In addition, previous studies have confirmed that obesity is closely related to the occurrence and development of RCC [23, 24]. A meta-analysis research found that BMI is associated with the risk of RCC [25]. However, Parker et al. [26] suggest that patients with high BMI in RCC had a lower degree of tumor malignancy. Our results showed that there was no significant correlation between BMI and pathological grades of ccRCC. Therefore, these contradictory results suggest that the relationship between obesity and RCC is complex and needs further study. Some studies showed that tumor growth patterns can predict pathological grades of ccRCC [15–18], but our study did not find a significant correlation between them, possibly due to the small sample size.

An important new finding of this study is that the presence of ARA in the affected kidney is an independent risk factor for high-grade ccRCC, which has not been reported in previous studies. Moreover, AUC showed that the multivariate prediction model of ARA combined with tumor maximum diameter could better predict high-grade ccRCC. At present, the mechanism of RAV and its effect on the pathological grade of RCC are still unclear. A study [27] performed in 2014 showed that in the embryonic kidney, several possible progenitor cells express the transcription factor Foxd1 precursor. Foxd1 itself regulates the origin, number, orientation, and cellular composition of renal vessels. Multiple accessory and aberrant arteries that originate from the abdominal aorta and iliac arteries likely cause the kidneys’ failure to ascend. Whether mutations of the Foxd1 gene in humans are associated with accessory or aberrant renal arteries remains to be investigated. In addition, Foxd1 is found to inhibit the ectopic differentiation and composition of the renal vascular system, but the molecular mechanisms involved need to be further studied.

The limitations of this study are as follows: (1) this study was a retrospective study, which might lead to selection bias although strict screening had been carried out; (2) this study was a single-center study with a small sample size, and the number of patients with high-grade ccRCC was small; and (3) only patients with ccRCC were included in this study, without comparison with normal population.

## Conclusion

In patients with ccRCC, the incidence of RAV in the affected kidney was significantly higher than that in the healthy kidney due to the influence of ARA. Importantly, this study found for the first time that ARA could be used as an independent predictor for Fuhrman grades of ccRCC. The finding provides a reference for the future diagnostic evaluation of RCC, and provides a new direction for the study of the pathogenesis of RCC. More prospective clinical studies with a large sample size, as well as relevant basic research, are needed in the future to explore the underlying biological mechanisms by which ARA affects the Fuhrman grade of ccRCC.

## Data Availability

The data that support the findings of the research are available from the corresponding author upon rational request.

## Abbreviations

RCC: Renal cell carcinoma
ccRCC: Clear cell RCC
RAV: Renal artery variation
ARA: Accessory renal artery
CT: Computed tomography
3D: Three-dimensional
BMI: Body mass index
ROC: Receiver operating characteristic
